# Multi-omics analysis reveals the molecular changes accompanying heavy-grazing-induced dwarfing of *Stipa grandis*

**DOI:** 10.3389/fpls.2022.995074

**Published:** 2022-10-04

**Authors:** Dongli Wan, Yongqing Wan, Tongrui Zhang, Ruigang Wang, Yong Ding

**Affiliations:** ^1^Institute of Grassland Research, Chinese Academy of Agricultural Sciences, Hohhot, China; ^2^College of Life Sciences, Inner Mongolia Agricultural University, Hohhot, China

**Keywords:** *Stipa grandis*, heavy grazing, transcriptomic, proteomic, metabolic

## Abstract

Heavy grazing significantly reduces *Stipa grandis* growth. To enhance our understanding of plant responses to heavy grazing, we conducted transcriptomic, proteomic, and metabolic analyses of the leaves of non-grazed plants (NG) and heavy-grazing-induced dwarf plants (HG) of *S. grandis*. A total of 101 metabolites, 167 proteins, and 1,268 genes differed in abundance between the HG and NG groups. Analysis of Kyoto Encyclopedia of Genes and Genomes pathways among differentially accumulated metabolites (DAMs) revealed that the most enriched pathways were flavone and flavonol biosynthesis, tryptophan metabolism, and phenylpropanoid biosynthesis. An integrative analysis of differentially expressed genes (DEGs) and proteins, and DAMs in these three pathways was performed. Heavy-grazing-induced dwarfism decreased the accumulation of DAMs enriched in phenylpropanoid biosynthesis, among which four DAMs were associated with lignin biosynthesis. In contrast, all DAMs enriched in flavone and flavonol biosynthesis and tryptophan metabolism showed increased accumulation in HG compared with NG plants. Among the DAMs enriched in tryptophan metabolism, three were involved in tryptophan-dependent IAA biosynthesis. Some of the DEGs and proteins enriched in these pathways showed different expression trends. The results indicated that these pathways play important roles in the regulation of growth and grazing-associated stress adaptions of *S. grandis*. This study enriches the knowledge of the mechanism of heavy-grazing-induced growth inhibition of *S. grandis* and provides valuable information for restoration of the productivity in degraded grassland.

## Introduction

*Stipa grandis* (Poaceae, 2*n* = 44) is a wind-pollinated C_3_ perennial bunchgrass that flowers in mid- to late-July and the seeds ripen in late August or early September (Zhao et al., 2008; Wu et al., 2010b). The mature plants form dense tussocks that are approximately 30 cm high with long and thin leaves (Zhao et al., 2008). *S. grandis* is among the dominant plant species in typical steppe of the Inner Mongolian Plateau (Wu et al., 2010b). *S. grandis*-dominated grassland, the most common and representative community in the Eurasian steppe (Gao et al., 2018b), has been degraded to varying degrees (Xiao et al., 1995; Wu et al., 2010a), which greatly affects grassland productivity.

Overgrazing and climate change (such as aridification) are the main factors responsible for degradation of grassland of the Inner Mongolian Plateau (Gao et al., 2018a). Livestock grazing is a major anthropogenic disturbance of grasslands on a global scale (Zhao et al., 2009). To respond to grazing, plants alter their morphological and functional traits (Zhao et al., 2009). For instance, the individual plant height and biomass (of leaves, stems, and the whole plant) of *Leymus chinensis* are significantly restricted under overgrazing (Li et al., 2015). The size of individual *S. grandis* plants is minimized to cope with frequent grazing or overgrazing (Wan et al., 2015; Li et al., 2018). The leaf photosynthetic activity, as indicated by net photosynthetic rate, stomatal conductance, intercellular carbon dioxide concentration, and transpiration rate, is markedly decreased following overgrazing (Ren et al., 2017). In recent years, several studies have been conducted to explore the molecular mechanism of plant dwarfism resulting from overgrazing at the transcription and protein levels (Wan et al., 2015; Ren et al., 2018a,b). However, little information is available on the protein and metabolic mechanisms responsible for the response of *S. grandis* to overgrazing.

Proteins are macromolecular compounds that function as the biochemical units in all cellular processes (Mann et al., 2013). Proteomics is the large-scale study of proteins and is crucial for understanding biological processes at the molecular level (Pandey and Mann, 2000; Zhang et al., 2014). Proteomics is widely used to study the processes of plant development and stress responses (Chen et al., 2015; Liu et al., 2015; Jiang et al., 2019; Zhan et al., 2019; Li et al., 2020; Lv et al., 2021), and provides essential tools to study global protein expression and dissect the unique functions underlying the many plant-specific biological processes (Job et al., 2011).

Plant metabolites, which are essential for humans as a nutritional source, play vital roles in the interaction of plants and the surrounding environment (Chen et al., 2020). It has been estimated that 200,000 to 1,000,000 metabolites exist in the plant kingdom (Saito and Matsuda, 2010). Metabolomics, which is the analysis of almost all metabolites in a biological sample using qualitative and quantitative methods, acts as a bridge to link the genome and phenome (Shi et al., 2020). Metabolomic approaches have been used to study metabolism in plant developmental processes and responses to environmental stimuli, such as low temperature (Yang et al., 2019b), salt (Li and Song, 2019), high light (Zhang et al., 2019), fruit development and ripening (Xu et al., 2020), and color variation (Yang et al., 2019a; Li et al., 2021b). Combined analysis of omics data has become a powerful tool to explain diverse developmental processes, environmental responses, and regulatory mechanisms (Chen et al., 2021; Guo et al., 2021; Xu et al., 2021; Li et al., 2021a; Qin et al., 2022). For instance, comparative transcriptomic and metabolomic analysis revealed an abscisic acid-dependent acclimation mechanism to drought and cold stress in maize (Guo et al., 2021). Combined transcriptomic, proteomic, and metabolomic analyses revealed that core metabolic processes were influenced in seeds of transgenic maize engineered for enhanced carotenoid synthesis (Decourcelle et al., 2015). Multi-omics analysis has highlighted distinct roles of central carbon metabolism to assist high productivity of specialized metabolites in glandular trichomes of tomato (Balcke et al., 2017), uncovered sequential roles associated with abscisic acid during seed maturation (Chauffour et al., 2019), and provided new insights into the decline in fruit quality of apple grown under high nitrogen fertilization (Wang et al., 2021b).

In this study, we performed transcriptomic, tandem mass tag (TMT) label-based proteomic, and widely targeted metabolic profiling in leaves of *S. grandis* grown under non-grazing and heavy-grazing treatments. The important metabolic pathways involved in the grazing response were evaluated by combined analysis of the omics data. The aim of this study was to elucidate the mechanism by which heavy grazing depresses *S. grandis* growth from different molecular aspects. The results will improve our understanding of the impact of grazing on metabolite and protein accumulation in *S. grandis*, and provide valuable information for restoration of productivity in degraded grassland.

## Materials and methods

### Study area and sampling

Sampling was conducted at the Inner Mongolia Typical Grassland Ecological Protection and Restoration Research Station of the Chinese Academy of Agricultural Sciences, located in Xilinhot, Inner Mongolia, China (116°32′E, 44°15′N), on August 2, 2018. The study region has a temperate semiarid continental climate, with a mean annual temperature of 0.7°C, and lowest and highest temperatures of −41.1°C in January and 38.5°C in August, respectively (Liu et al., 2019). The mean annual precipitation is 300–360 mm with maximum precipitation in the period from June to August (Zhang et al., 2020). The dominant plant species in the study area comprised *S. grandis* and *L. chinensis*.

Grazing had been prohibited from the study area from 2007 to 2013, and the grazing experiments were conducted since 2014. The non-grazed (NG) plot was continually protected from grazing from 2014 to 2018. The heavy-grazing (HG) plot was grazed from 2014 to 2017 by 12 sheep. Grazing started in the middle of June and ended in the middle of September each year. The NG and HG plots were 1.33 Ha in area. The grazing pressure of HG is 1.4 standard sheep unit per hectare per year (SSU·ha^−1^·y^−1^), which is apparently higher than that of locally allowed standards (0.84 SSU·ha^−1^·y^−1^). Each grazing treatment was established with three replicate plots. In 2018, prior to grazing, half of each HG plot was fenced to exclude grazing disturbance, and the individuals of *S. grandis* in this area retained a dwarf phenotype compared with the plants in the NG area (Supplementary Figure S1); the HG samples for subsequent analysis were collected from plants growing in the exclusion area. The leaves from three bunches of *S. grandis*, sampled at a vigorous growth stage from one plot for each treatment, were respectively pooled as one biological replicate, immediately frozen in liquid nitrogen, and stored at −80°C for subsequent total RNA, protein, and metabolite extraction. Three biological replicates were taken from one sampling plot for each treatment.

### Transcriptome analysis

Total RNA of each sample was isolated from leaves of three bunches of *S. grandis* using TRIzol Reagent. The RNA integrity was examined using agarose gel electrophoresis and an Agilent 2,100 Bioanalyzer. The RNA concentration and purity were determined with a NanoDrop spectrophotometer based on the OD_260_/OD_280_ and OD_260_/OD_230_ ratios, respectively.

For cDNA library construction, mRNAs were enriched from the total RNAs using Oligo (dT) magnetic beads, after fragmentation into short segments using fragmentation buffer, used as templates to synthesize the first-strand cDNA with random hexamer primers and reverse transcriptase. Following second-strand cDNA synthesis, the fragments were end-repaired, a single adenine nucleotide was added to the 3′ end, and then ligated with sequencing adapters. Subsequently, the cDNA fragments were selected and PCR amplification was performed to obtain the final cDNA library. In total, six libraries were sequenced using an Illumina NovaSeq 6,000 platform.

Following processing for quality control, transcriptome assembly was performed with Trinity (Grabherr et al., 2011). Gene functions were annotated using the Kyoto Encyclopedia of Genes and Genomes (KEGG),^1^ Gene Ontology (GO),^2^ National Center for Biotechnology Information (NCBI) non-redundant (NR),^3^ Swiss-Prot,^4^ UniProtKB/TrEMBL,^5^ and Clusters of Orthologous Groups of proteins (COG**/**KOG)^6^ databases. The transcript levels of unigenes were calculated using the fragments per kilobase per million fragments (FPKM) method. Differential expression analysis between the HG and NG treatments was performed using DESeq2 (Love et al., 2014; Varet et al., 2016). The significantly differentially expressed genes (DEGs) were determined with the thresholds of |log_2_ (HG/NG)| ≥ 1 and FDR ≤ 0.05. Analyses of GO term and KEGG pathway enrichment of the DEGs were conducted with the R version 3.5.1 software using R package “clusterProfiler” (Yu et al., 2012) using the threshold of a corrected value of *p* ≤ 0.05 to determine significant enrichment.

### Proteome analysis

#### Protein extraction and TMT labeling

Protein extraction from leaves of each sample for proteomic analysis was performed following a previously described method (Wang et al., 2021a). The protein concentration was calculated using the Bradford assay with bovine serum albumin as the standard (Bradford, 1976). Protein quality was examined using SDS-PAGE. The proteins extracted from each sample were digested with Trypsin Gold (Promega, Madison, WI, United States) at 37°C for 16 h. The peptide was desalted using a C18 cartridge and dried by vacuum centrifugation. The TMT labeling of peptides was performed using TMTsixplex reagents (TMTsixplex^™^ Isobaric Label Reagent Set, Thermo Fisher, Waltham, MA, Unites States) in accordance with the manufacturer’s instructions.

#### HPLC fractionation and LC–MS/MS analysis

Fractionation of the TMT-labeled peptide mix was performed on a Rigol L3000 HPLC using a C18 column (4.6 mm × 250 mm, 5 μm), with a column temperature of 50°C. Mobile phases A and B were water solutions containing 2% and 98% acetonitrile (pH 10.0), respectively. The TMT-labeled peptide mix was dissolved in 1 ml A and the supernatant was used as the injection sample. The peptides were separated into 15 fractions based on the following elution gradient: 3% B, 5 min; 3–8% B, 0.1 min; 8–18% B, 11.9 min; 18–32% B, 11 min; 32–45% B, 7 min; 45–80% B, 3 min; 80% B, 5 min; 80%–5% B, 0.1 min; and 5% B, 6.9 min. Following drying under vacuum, the samples were reconstituted in 0.1% (v/v) formic acid (FA) in water and used for LC–MS/MS analyses.

For proteomic analyses, the EASY-nLC™ 1200 UHPLC system (Thermo Fisher) was employed to analyze the samples. A Q Exactive^™^ HF-X Orbitrap mass spectrometer (Thermo Fisher) was used to obtain the peptides in the data-dependent acquisition (DDA) mode. Briefly, 2 μg peptides were dissolved in 0.1% FA and injected into a C18 Nano-Trap column (2 cm × 100 μm, 5 μm). The peptides were separated with a ReproSil-Pur 120 C18-AQ analytical column (15 cm × 150 μm, 1.9 μm), with a linear gradient from 5% to 100% eluent B (0.1% FA in 80% acetonitrile) in eluent A (0.1% FA in H_2_O) for 90 min with a flow rate of 600 nl/min.

For DDA, the MS conditions were set as follows: spray voltage 2.3 kV, capillary temperature 320°C, range of full MS scans 350–1,500 *m/z*, resolution 60,000 (200 *m/z*), automatic gain control (AGC) target value 3 × 10^6^, and maximum ion injection time 20 ms. Based on the full MS scan, the 40 most abundant precursor ions were selected for higher-energy collisional dissociation fragment analysis. Resolution was set to 15,000, with AGC target value 1 × 10^5^, maximum ion injection time 45 ms, normalized collision energy of peptide fragmentation 32%, intensity threshold 8.3 × 10^3^, and dynamic exclusion parameter 60 s.

#### Protein identification and bioinformatics analysis

For protein identification, Proteome Discoverer (version 2.2) was used to search the raw data derived from MS analysis against the *S. grandis* transcriptome database (NCBI Sequence Read Archive database: PRJNA867365). The reporter quantification type was set as TMTsixplex. Trypsin was specified as an enzyme with a maximum of two missed cleavage sites. Mass tolerance for precursor and fragment was 10 ppm and 0.02 Da, respectively. Dynamic modifications were set as oxidation of methionine and TMTsixplex labeling lysine. The fixed modification was set as carbamidomethyl on cysteine. The parameters used for N-terminal modifications were acetylation of peptide N-terminus and TMTsixplex labeling N-terminus. Peptides were quantified according to the peak areas of corresponding mass reporters. The sequence of the peptides was determined by the mass-to-charge ratio of the peptide fragment ion peak. To reduce the frequency of false positives, the searched data were subsequently filtered through Proteome Discoverer. A peptide–spectrum match of 95% confidence was identified as credible, and the proteins containing at least one unique peptide were identified as credible. The false discovery rate (FDR) value was set at <5%.

Protein annotation was conducted using the GO, KEGG, and COG databases. For TMT quantification of proteins, the fold change (FC) between HG and NG samples was determined from the mean ratios of the TMT reporter ion intensities in the MS/MS spectra (*m/z* 126, 127-130C, 131) from all raw data sets. The significance of the differences was calculated using the corresponding value of *p* of each protein in two compared samples determined using Student’s *t*-test. The thresholds of FC ≥ 1.2 or FC ≤ 0.83 and value of *p* ≤ 0.05 were applied to detect differentially expressed proteins (DEPs). The functional subgroups and metabolic pathway enrichment for the DEPs were determined using the GO and KEGG databases.

### Metabolite extraction and profiling

Metabolite extraction and profiling of the HG and NG samples was conducted by Wuhan Metware Biotechnology Co., Ltd. (Wuhan, China). Metabolite extraction, metabolite data acquisition, and assessment followed standard protocols as described in full previously (Chen et al., 2013; Li et al., 2021a). Briefly, the freeze-dried leaves were ground to powder using a mixer mill (MM 400, Retsch) with a zirconia bead (30 Hz, 1.5 min). One hundred milligram powder was extracted with 1.0 ml 70% aqueous methanol overnight at 4°C. After centrifugation (10,000 *g*, 10 min), the supernatant was filtrated using a micropore filter membrane (0.22 μm pore size) for LC–MS analysis. A LC-ESI-MS/MS system (UPLC, Shim-pack UFLC SHIMADZU CBM30A system, Kyoto, Japan; MS, Applied Biosystems 6,500 QTRAP, Framingham, MA, USA) was used for the UPLC–MS analysis. The chromatographic conditions were as follows: column, Waters ACQUITY UPLC HSS T3 C18 (1.8 μm, 2.1 mm × 100 mm); solvent system, water (0.04% acetic acid), acetonitrile (0.04% acetic acid); elution gradient, water: acetonitrile was 95:5 (V/V) at 0 min, 5:95 (V/V) at 11.0 and 12.0 min, 95:5 (V/V) at 12.1 and 15.0 min; flow rate 0.4 ml min^−1^; column temperature 40°C, sample injection volume 2 μl. The mass spectrometry conditions: ESI temperature 500°C; ion spray voltage 5,500 V, curtain gas 25 psi; collision-activated dissociation was set to high. Instrument tuning was performed with 10 μmol/l polypropylene glycol solutions in triple quadrupole mode, and mass calibration was performed with 100 μmol/l polypropylene glycol solutions in linear ion trap mode. Metabolite identification was performed using the MWDB database.^7^ Quantitative metabolite analysis was conducted using the multiple reaction monitoring mode (Fraga et al., 2010).

Pearson’s correlation analysis was used to analyze the correlation among samples within a group to assess biological repeatability. Hierarchical cluster analysis was conducted with R software^8^ to determine the metabolite accumulation patterns among different samples. Unsupervised principal component analysis (PCA) was employed to analyze the variability between the HG and NG groups as well as the three replications in each group.

The metabolite FC was calculated as the ratio of the mean metabolite abundance of HG plants relative to NG plants. The differentially accumulated metabolites (DAMs) were determined by a combination of the FC values and variable influence on projection (VIP) values. Partial least squares–discriminant analysis (PLS-DA), which is a supervised multivariate method, was used to maximize the metabolome differences between a pair of samples. The VIP value was used to check the relative importance of each metabolite in the PLS-DA model. Metabolites with VIP ≥ 1 and FC ≥ 2 or FC ≤ 0.5 were identified as significantly differentially accumulated. The KEGG database was accessed for metabolite classification and pathway enrichment analysis.

### Weighted correlation network analysis

Co-expression networks of DAMs and genes were constructed with R version 3.5.1 software using the weighted gene co-expression network analysis (WGCNA) package (version1.69), the parameter were set as follow: powerEstimate18, mergeCutHeight 0.25, and minModuleSize 50.

## Results

### Transcriptome profile of *Stipa grandis*

**Figure 1:**
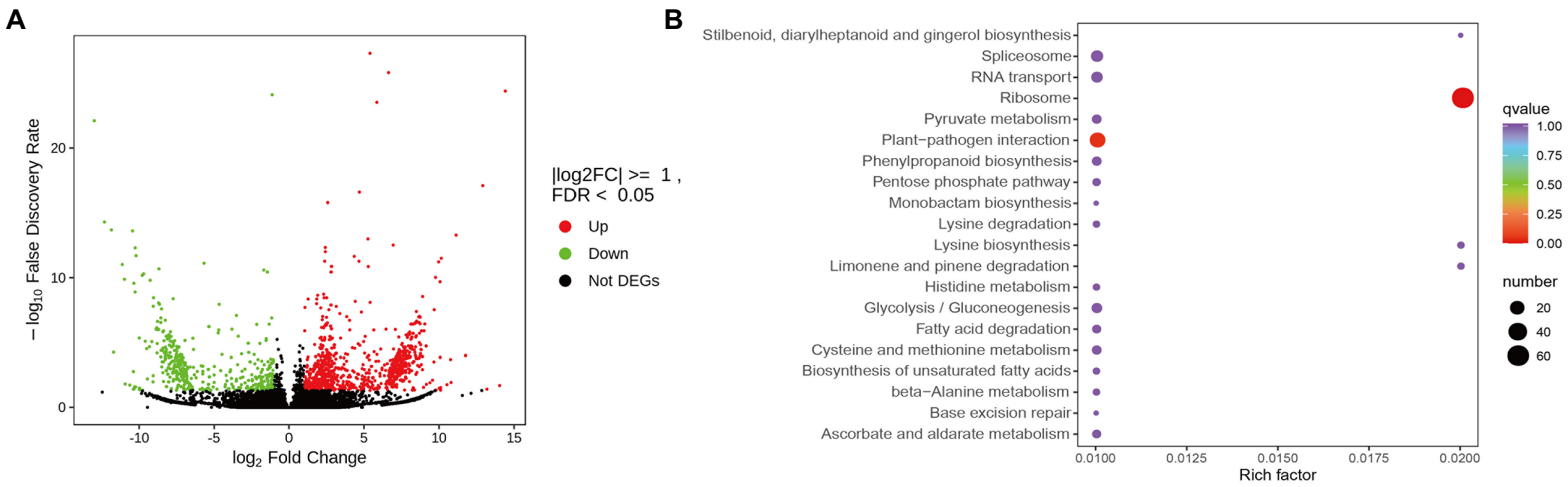
Differentially expressed genes (DEGs) between non-grazed (NG) and heavy-grazing (HG) plants of *Stipa grandis*. **(A)** Heatmap analysis of DEGs. **(B)** KEGG pathway enrichment analysis of DEGs.

To investigate the molecular basis of the impact of heavy grazing on *S. grandis*, transcriptome profiling was performed and the transcript abundances were compared between the HG and NG samples (Supplementary Table S1). In total, 1,268 DEGs were detected, of which 860 were up-regulated and 408 were down-regulated (Figure 1A; Supplementary Table S2). Seventy-two GO terms were significantly enriched among the DEGs (Supplementary Figure S2). The most enriched KEGG pathways among these DEGs were the ribosome, plant–pathogen interaction, limonene and pinene degradation, lysine biosynthesis, ascorbate and aldarate metabolism, stilbenoid, diarylheptanoid, and gingerol biosynthesis; and histidine metabolism pathways (Figure 1B). The ribosome and plant–pathogen interaction pathways were significantly enriched (*p* ≤ 0.05; Figure 1B).

### TMT label-based proteome profile of *Stipa grandis*

**Figure 2:**
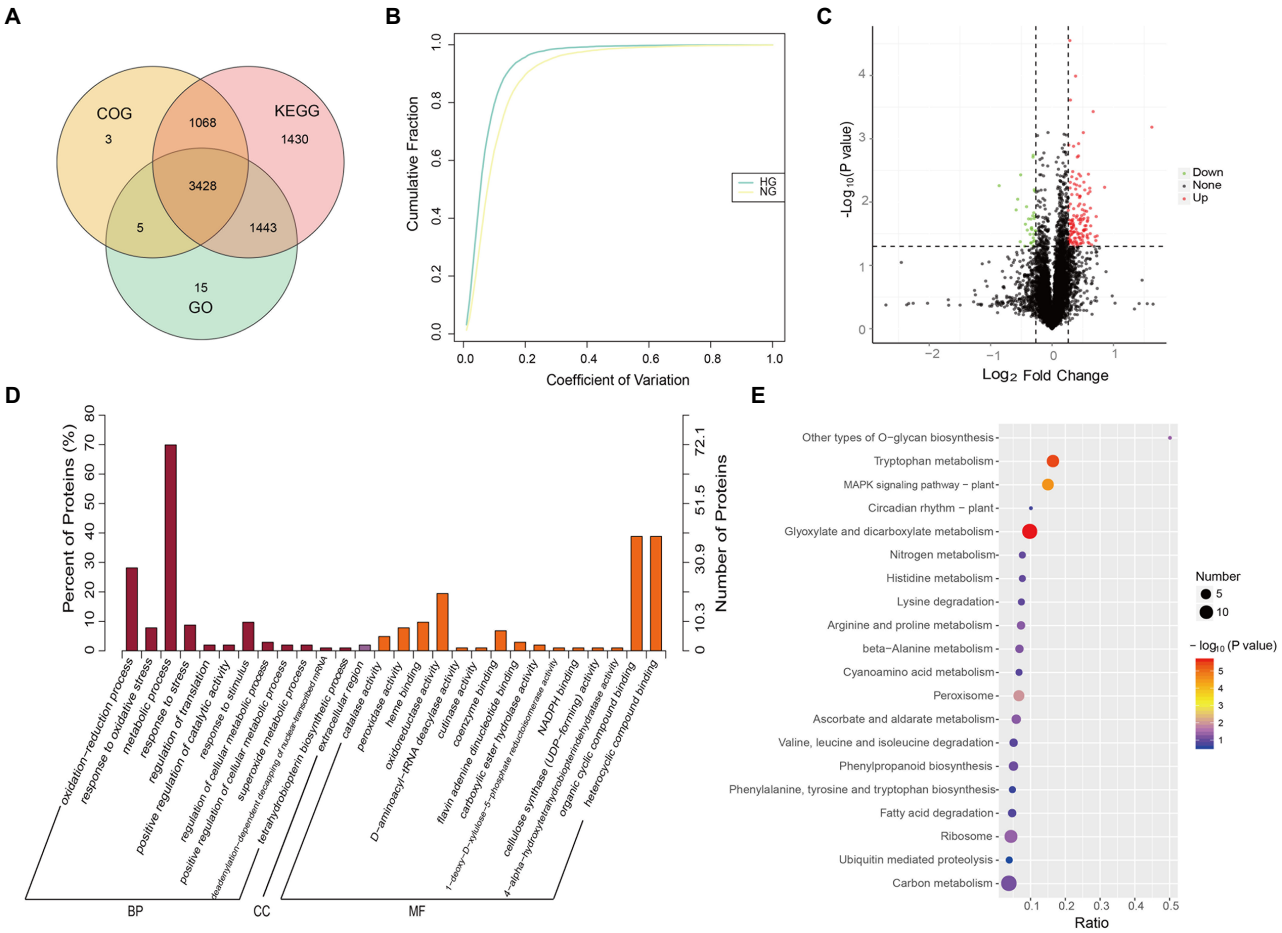
Proteome profile and differentially expressed proteins (DEPs) between NG and HG plants of *S. grandis*. **(A)** Venn diagram of proteins annotated from the GO, KEGG, and COG databases. **(B)** Coefficient of variation cumulative curve for NG and HG samples. **(C)** Quantitative analysis of the proteome of NG and HG samples. Green, red, and black represent down-regulated, up-regulated, and non-significantly changed proteins, respectively. **(D)** GO term enrichment analysis of DEPs between NG and HG samples. BP, biological process; CC, cellular component; and MF, molecular function. **(E)** KEGG pathway enrichment analysis of DEPs between NG and HG samples.

The proteomic differences between the HG and NG groups were analyzed using TMT-based LC–MS/MS. Detailed information on the quality control is presented in Supplementary Figure S3. In total, 7,456 proteins were detected, of which 7,392 were annotated with functional terms from at least one of the GO, KEGG, and COG databases (Figure 2A; Supplementary Table S3). The coefficient of variation for most proteins was less than 20% (Figure 2B), which demonstrated high repeatability among the three biological replicates in each HG and NG group.

The abundance of 167 proteins differed significantly between the HG and NG groups with the thresholds FC ≥ 1.2 or FC ≤ 0.83, and value of *p* ≤ 0.05. The expression of 135 DEPs was upregulated, whereas that of 32 DEPs was downregulated, in HG compared with NG (Figure 2C). Functional enrichment analysis showed that the DEPs were significantly enriched with 28 GO terms (*p* ≤ 0.05, Figure 2D; Supplementary Table S4). The five most enriched terms in the molecular function category were catalase activity, peroxidase activity, heme binding, oxidoreductase activity, and D-aminoacyl-tRNA deacylase activity. The five most enriched terms associated with the biological process category were oxidation–reduction process, response to oxidative stress, metabolic process, response to stress, and regulation of translation. Only one enriched term, extracellular region, was associated with the cellular component category. The KEGG pathway analysis showed that the DEPs were enriched with 36 pathways (Figure 2E; Supplementary Table S4). It was noteworthy that glyoxylate and dicarboxylate metabolism, tryptophan metabolism, MAPK signaling pathway-plant, peroxisome, other types of *O*-glycan biosynthesis, and ribosome pathway were significantly enriched (*p* ≤ 0.05).

### Widely targeted metabolic profile analysis of *Stipa grandis*

**Figure 3:**
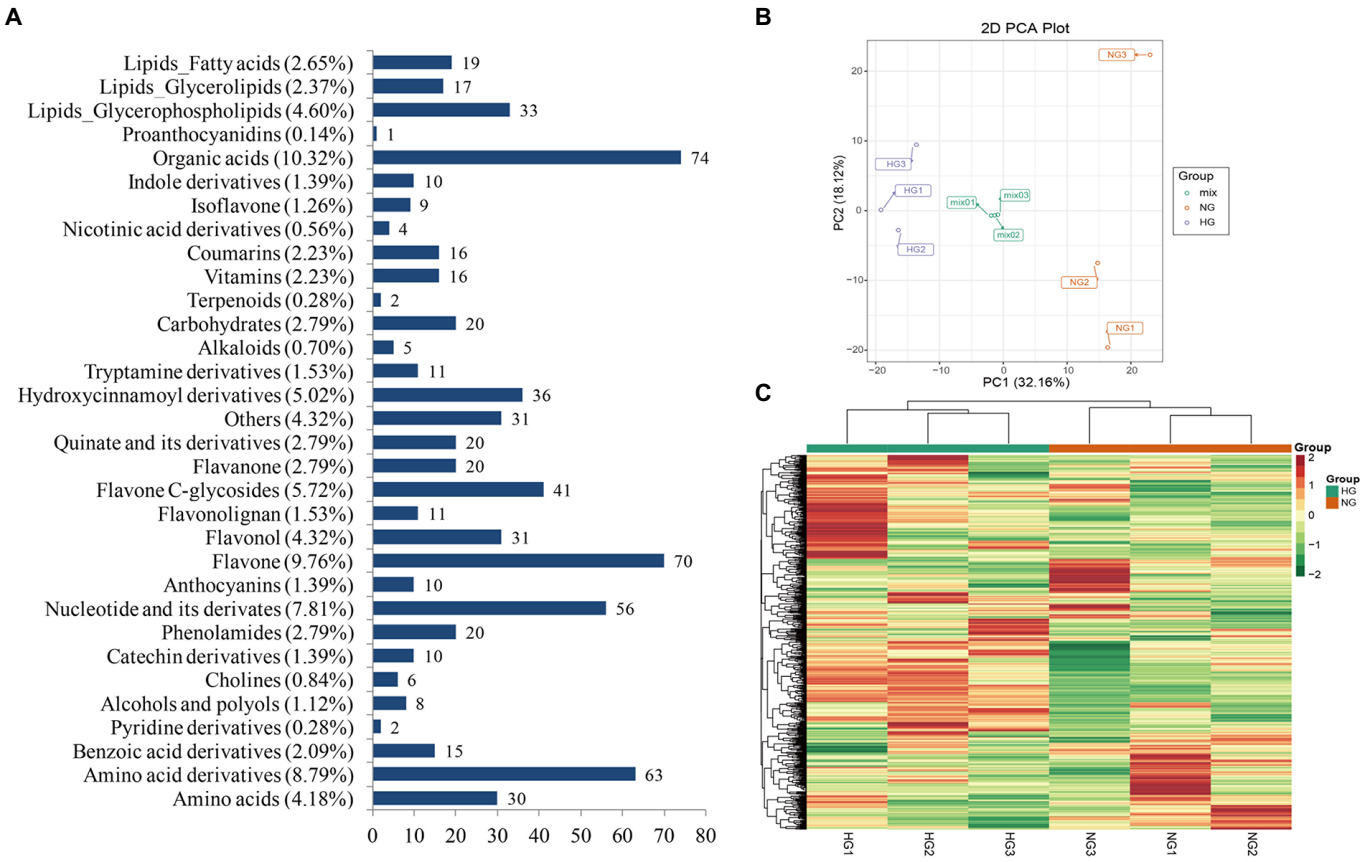
Qualitative and quantitative analysis of metabolites in leaves of *S. grandis*. **(A)** Classification of all identified metabolites. **(B)** Principal component analysis scatterplot of leaf samples from NG and HG plants. **(C)** Cluster analysis heatmap of all identified metabolites. The color scale indicates the abundance of each metabolite.

To investigate the impact of heavy grazing on metabolic changes in *S. grandis*, the metabolome was detected in leaves from HG and NG samples of *S. grandis* by LC–MS/MS analysis. In total, 717 metabolites were identified (Supplementary Table S5). The metabolites were assigned to 32 types (Figure 3A). The five most abundant metabolite types were organic acids (10.32%), flavones (9.76%), amino acid derivatives (8.79%), nucleotides and derivatives (7.81%), and flavone C-glycosides (5.72%). The PCA revealed a clear separation between HG and NG plants (Figure 3B). Similarly, the heatmap cluster analysis clearly divided the six samples into two groups (Figure 3C). In addition, the Pearson’s correlation analysis showed that the intragroup samples were significantly correlated (Supplementary Figure S4).

**Figure 4:**
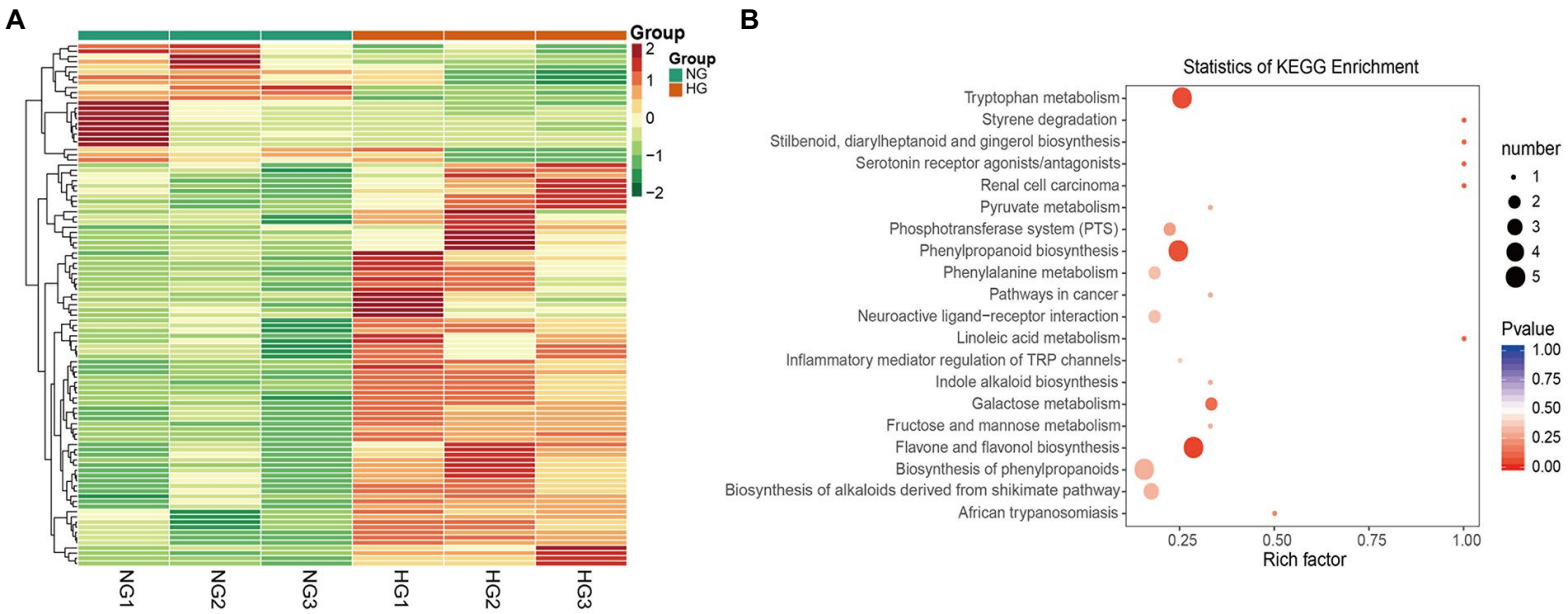
Differentially accumulated metabolites (DAMs) between NG and HG plants of *S. grandis*. **(A)** Cluster analysis heatmap of DAMs. **(B)** KEGG pathway enrichment analysis of DAMs.

A total of 101 metabolites differed significantly between the HG and NG groups (Figure 4A). The metabolites were classified into 21 categories, and comprised 78 upregulated and 23 downregulated metabolites (Table 1). The top 20 DAMs were mainly associated with flavones, flavone C-glycosides, quinate and its derivatives, catechin derivatives, carbohydrates, benzoic acid derivatives, organic acids, indole derivatives, hydroxycinnamoyl derivatives, amino acid derivatives, alcohols and polyols, and phenolamides (Supplementary Figure S5).

**Table 1:** Classification of significantly differentially accumulated metabolites between NG and HG plants of *S. grandis*.

Class	Total	Up-regulation	Down-regulation
Amino acid derivatives	8	7	1
Benzoic acid derivatives	3	2	1
Alcohols and polyols	3	3	0
Catechin derivatives	2	1	1
Phenolamides	3	3	0
Nucleotide and its derivates	2	0	2
Anthocyanins	1	1	0
Flavone	22	22	0
Flavonol	6	6	0
Flavone C-glycosides	17	17	0
Flavanone	2	2	0
Quinate and its derivatives	4	0	4
Others	2	0	2
Hydroxycinnamoyl derivatives	4	0	4
Tryptamine derivatives	4	4	0
Carbohydrates	3	1	2
Indole derivatives	1	1	0
Organic acids	5	1	4
Lipids_Glycerophospholipids	5	3	2
Lipids_Glycerolipids	1	1	0
Lipids_Fatty acids	3	3	0

Based on KEGG pathway classification, the DAMs were involved in 64 pathways (Supplementary Table S6). The ten most enriched metabolic pathways were (in decreasing order) flavone and flavonol biosynthesis, tryptophan (Trp) metabolism, phenylpropanoid biosynthesis, stilbenoid, diarylheptanoid, and gingerol biosynthesis, styrene degradation, linoleic acid metabolism, renal cell carcinoma, serotonin receptor agonists/antagonists, galactose metabolism, and African trypanosomiasis (Figure 4B). Notably, the flavone and flavonol biosynthesis pathway was significantly enriched (*p <* 0.05) in the comparison of HG and NG.

### KEGG pathways shared among DEGs, DEPs, and DAMs

**Figure 5:**
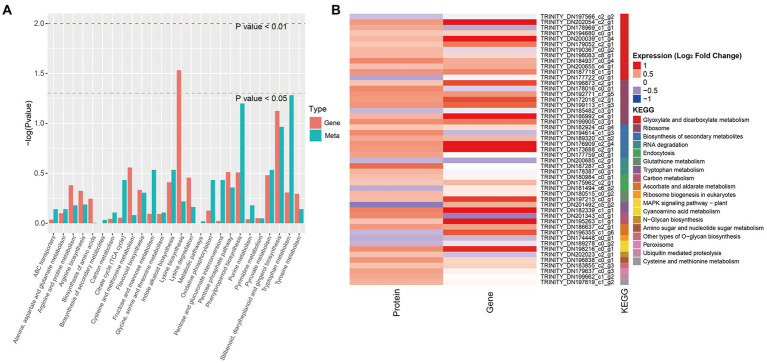
Enriched KEGG pathways common to DAMs, DEPs, and DEGs between NG and HG plants of *S. grandis*. **(A)** Histogram of KEGG pathways common to DAMs and DEGs. **(B)** Heatmap of KEGG pathways common to DEPs and DEGs.

The KEGG pathway enrichment analysis of the DEGs and DAMs showed that 26 KEGG pathways were shared among the significantly changed metabolites and transcripts (Figure 5A). Relatively high enrichment was observed among the stilbenoid, diarylheptanoid and gingerol biosynthesis, phenylpropanoid biosynthesis, tryptophan metabolism, pyruvate metabolism, degradation of aromatic compounds, indole alkaloid biosynthesis, pentose phosphate, and flavonoid biosynthesis pathways. Combined analysis of KEGG pathway enrichment among DEGs and DEPs revealed that the top shared pathways were glyoxylate and dicarboxylate metabolism, ribosome, biosynthesis of secondary metabolites, RNA degradation, and endocytosis (Figure 5B).

### Joint analysis of phenylpropanoid biosynthesis, and flavone and flavonol biosynthesis

**Figure 6:**
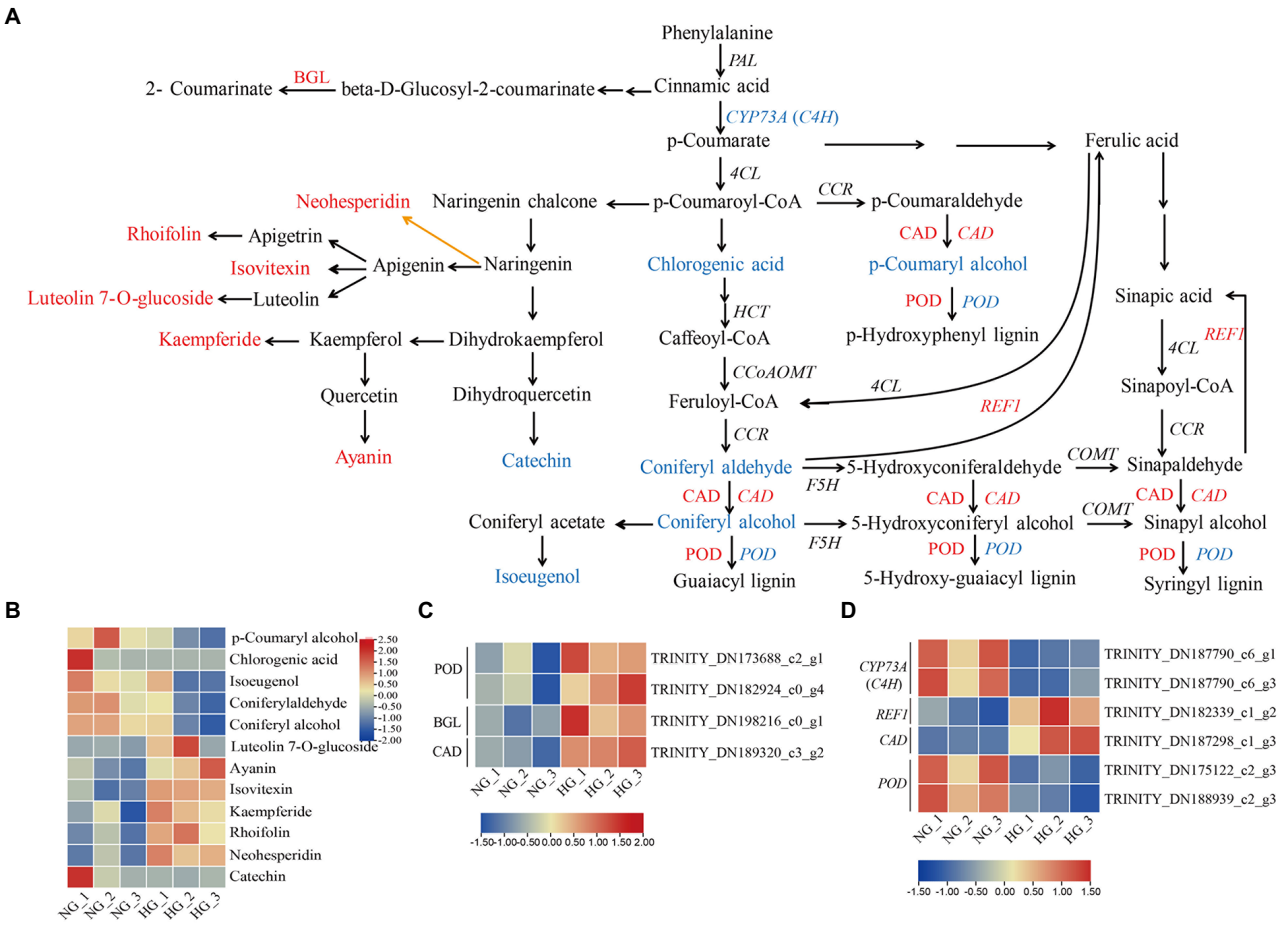
Phenylpropanoid biosynthesis, and flavone and flavonol biosynthesis pathways in *S. grandis* in response to heavy grazing. **(A)** Joint analysis of the DAMs, DEPs, and DEGs. Red and blue letters represent up- and down-regulation, respectively. Upper-case letters indicate proteins and italicized upper-case letters indicate genes. Orange line indicate biosynthetic step is not clear. The heatmap represents the corresponding expression levels of **(B)** DAMs, **(C)** DEPs, and **(D)** DEGs in the NG and HG groups. The color scale indicates the relative expression level.

Given that flavone and flavonol biosynthesis as well as phenylpropanoid biosynthesis were among the three most highly enriched pathways in the metabolic profile analysis, an integrated analysis of DAMs, DEGs, and DEPs was performed (Figure 6). In the phenylpropanoid biosynthesis pathway, all of the five DAMs, namely *p*-coumaryl alcohol, chlorogenic acid, coniferylaldehyde, coniferyl alcohol, and isoeugenol, showed decreased accumulation in HG compared with NG samples. Four proteins, comprising two peroxidases (POD), one beta-glucosidase (BGL), and one cinnamyl-alcohol dehydrogenase (CAD), showed enhanced expression in HG compared with NG leaves. Six genes were differentially expressed in HG compared with the NG group, among which two *trans*-cinnamate 4-monooxygenase-encoding genes (*CYP73A*) and two POD-encoding genes were down-regulated, and one CAD-encoding gene and one cinnamyl-alcohol dehydrogenase-encoding gene (*REF1*) were up-regulated. In the flavone and flavonol biosynthesis pathway, five metabolites, namely luteolin 7-*O*-glucoside, ayanin, isovitexin, kaempferide, and rhoifolin, showed differential accumulation patterns between the HG and NG groups, with higher accumulation observed in HG than in NG samples. However, no enrichment of significant DEGs or DEPs was observed. In addition, two DAMs (neohesperidin and catechin) that were mapped to the flavonoid biosynthesis pathway showed altered accumulation in HG samples. WGCNA was performed to identify potential genes co-expression with these DAMs. The *CAD* and *REF1*were distributed to module of yellow, and negatively correlated with DAMs of phenylpropanoid biosynthesis; while *CYP73As* and *PODs* were distributed to module grey60, and positively correlated with DAMs of phenylpropanoid biosynthesis (Supplementary Figure S6).

### Joint analysis of Trp metabolism

**Figure 7:**
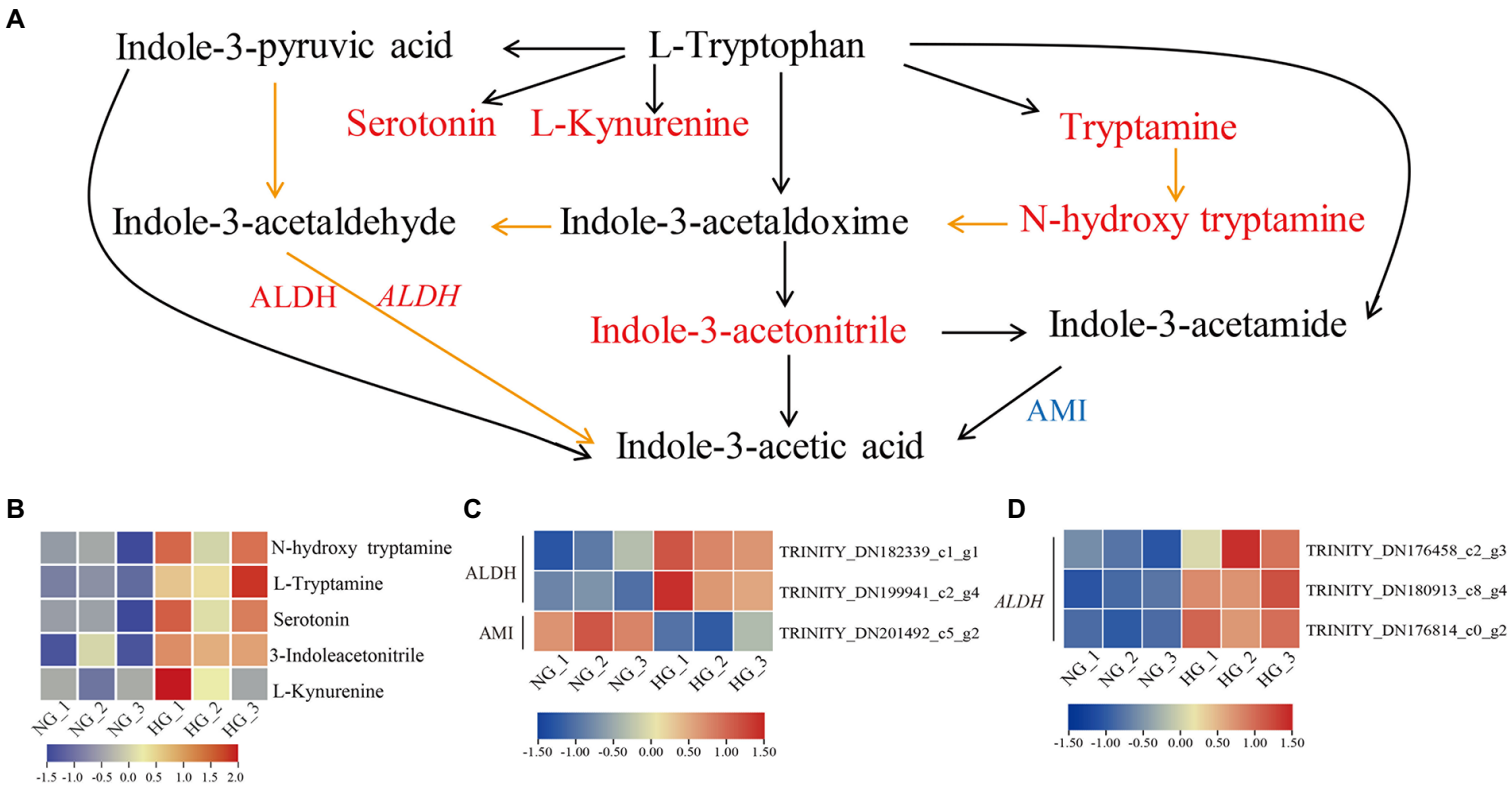
Tryptophan metabolism pathway in *S. grandis* in response to heavy grazing. **(A)** Joint analysis of the DAMs, DEPs, and DEGs. Red and blue letters represent up- and down-regulation, respectively. Upper-case letters indicate proteins and italicized upper-case letters indicate genes. Orange lines represent biosynthetic steps that are not clear. The heatmap represents the corresponding expression levels of **(B)** DAMs, **(C)** DEPs, and **(D)** DEGs in the NG and HG groups. The color scale indicates the relative expression level.

To evaluate the impact of heavy grazing on Trp metabolism, an integrated analysis of DAMs, DEPs, and DEGs associated with auxin biosynthesis was conducted (Figure 7). All five enriched metabolites involved in Trp metabolism were more highly accumulated in HG compared with NG samples, among which tryptamine (TAM), *N*-hydroxytryptamine, and indole-3-acetonitrile (IAN) are involved in Trp-dependent indole-3-acetic acid (IAA) biosynthesis. Three DEPs were associated with IAA biosynthesis, of which two aldehyde dehydrogenase (ALDH) family 2 proteins were up-regulated, and one amidase (AMI), which is involved in the conversion of indole-3-acetamide (IAM) into active IAA, was down-regulated in HG compared with NG samples. Three DEGs associated with *ALDH* genes were up-regulated in HG compared with NG samples. WGCNA analysis showed that one and two *ALDHs* distributed to yellow and green modules, respectively, and positively correlated with the DAMs of Trp metabolism (Supplementary Figure S7).

## Discussion

Grazing is the most widespread and important land use in Inner Mongolia. The plant morphology, grassland productivity, and vegetation dynamics are affected by different grazing intensities (Liu et al., 2019). In this study, we investigated the mechanism for heavy-grazing-induced plant dwarfism by conducting an integrated analysis of the transcriptome, proteome, and metabolome. Based on the metabolic profile analysis, 101 metabolites were significantly differentially accumulated (VIP ≥ 1) between the HG and NG groups. These DAMs were assigned to 64 KEGG pathways among which the three most enriched pathways were flavone and flavonol biosynthesis, tryptophan metabolism, and phenylpropanoid biosynthesis.

### Phenylpropanoid biosynthesis, and flavone and flavonol biosynthesis response to heavy grazing

The phenylpropanoid pathway is among the most extensively investigated specialized metabolic pathways (Dong and Lin, 2021). It gives rise to a vast variety of aromatic metabolites (Zhang and Liu, 2015). The lignin and flavonoid pathways are two major branches among the diverse branches of phenylpropanoid metabolism derived from the general phenylpropanoid pathway (Dong and Lin, 2021). In which, phenylalanine is catalyzed by phenylalanine ammonia lyase to form *trans*-cinnamic acid, which is hydroxylated by cinnamic acid 4-hydroxylase (C4H) to generate *p*-coumaric acid. In turn, *p*-coumaric acid is converted into *p*-coumaroyl-CoA catalyzed by 4-coumarate-CoA ligase (4CL; Dong and Lin, 2021).

Lignin, primarily a component of secondarily thickened cell walls, is essential for mechanical support during plant growth, facilitates the transportation of water and nutrients, and plays roles in plant resistance to pathogen attack (Boerjan et al., 2003). Lignin is derived mainly from three monolignols, namely *p*-coumaryl (M1_H_), coniferyl (M1_G_), and sinapyl (M1_S_) alcohols, which are used to produce the three basic units of lignin, *p*-hydroxyphenyl (H), guaiacyl (G), and syringyl (S), respectively (Boerjan et al., 2003; Dong and Lin, 2021). Alteration of monolignol metabolism strongly impacts on plant growth (Meents et al., 2018). However, the mechanism of dwarfism caused by suppression of monolignol biosynthesis is not completely elucidated, but several factors may contribute, such as water transport disruption, lack of cell proliferation or expansion resulting from failed synthesis of a phenylpropanoid-derived compound, or accumulation of a toxic pathway intermediate (Bonawitz and Chapple, 2010). In the present study, four of the five DAMs enriched in phenylpropanoid biosynthesis that were decreased in abundance in HG were involved in lignin biosynthesis, namely chlorogenic acid, coniferylaldehyde, and the monolignols coniferyl alcohol and *p*-coumaryl alcohol. Inconsistent with this finding, three enzymes were more highly accumulated in HG compared with NG. One of these enzymes was CAD, which functions in the final step of monolignol biosynthesis and is responsible for catalyzing the cinnamaldehydes to the corresponding alcohols, namely *p*-coumaryl alcohol, caffeyl alcohol, coniferyl alcohol, 5H coniferyl alcohol, and sinapyl alcohol (Boerjan et al., 2003; Dong and Lin, 2021). The other two enzymes were PODs, which are involved in the dehydrogenative polymerization of monolignols to form lignin (Fraser and Chapple, 2011). One CAD-encoding gene was up-regulated and two POD-encoding genes were downregulated. The inconsistent expression patterns of POD proteins and *POD* genes may result from the lower correlation between transcript and protein level due to post-transcriptional and post-translational regulations (Kumar et al., 2016; Xu et al., 2021). Alternatively, because these belong to a different member of POD family that may play different roles in lignin biosynthesis. C4H is a unique member of CYP73A, termed as CYP73A5 in *Arabidopsis thaliana* (Mizutani et al., 1997; Fraser and Chapple, 2011). Mutant of *C4H* displayed a dwarf phenotype in *A. thaliana* (Schilmiller et al., 2009). In the present study, down-regulated expression of two *C4H* members, i.e the *CYP73A* genes, in HG compared with NG, may be positively related to the heavy-grazing-induced dwarfism of *S. grandis*. Taken together, these results indicated that monolignol biosynthesis was inactivated in the heavy-grazing-induced dwarfism of *S. grandis*, and decreased production of monolignols may be associated with the plant morphological phenotype and reduction of lignin-mediated defense responses. But the regulatory mechanism of reduced monolignol biosynthesis associated with the dwarfism of *S. grandis* remains unclear. Additionally, the activated expression of proteins or genes in HG may be associated with restored promotion of growth following the exclusion of grazing after the spring regreening stage. Further research on the molecular regulation of monolignol biosynthesis activity to adapt to heavy grazing is necessary.

Flavonoids, a major class of phenolic compounds, possess a basic C6–C3–C6 skeleton and are divided into numerous subgroups, such as flavanones, flavanols, flavones, and flavonols (Nakayama et al., 2019; Nabavi et al., 2020). Flavonoids perform a wide range of roles in plant development and environmental adaptation (Peer and Murphy, 2007; Thompson et al., 2010; Hichri et al., 2011). In particular, flavonoid biosynthesis is induced by oxidative stress (Nabavi et al., 2020), and flavonoids act as antioxidants to decrease oxidative damage triggered by reactive oxygen species under exposure to stresses (Nakabayashi and Saito, 2015; Dong and Lin, 2021). In the present study, all five DAMs involved in flavone and flavonol biosynthesis were more highly accumulated in the HG group than in NG samples. Except for ayanin, which has antimicrobial activity (French et al., 1991), the other four metabolites, namely luteolin 7-*O*-glucoside, isovitexin, kaempferide, and rhoifolin, exhibit antioxidant capabilities (Picerno et al., 2003; Eldahshan and Azab, 2012; Bian et al., 2013; Lama-Muñoz et al., 2019). This finding indicated that heavy-grazing-induced dwarfism may enhance tolerance to environmental stresses and might be mediated by flavonoid compounds through decreased oxidative damage. However, little is known about the underlying regulatory mechanism. Grazing caused multi-stress, involved in trampling/wounding, defoliation, saliva deposition, and manure deposition (Chen et al., 2009). Over or heavy grazing resulted in grassland degradation, including but not limited to reduced vegetation coverage and species diversity, declined plant growth, decreased soil coverage and water conservation, as well as changed soil microbial structure (Han et al., 2008; Yang et al., 2016). Whether these factors contribute to the changed accumulation pattern of flavonoids in dwarf plants, and which is the driving factor, is worth further confirmation by conducting the single or cross-stress experiments in *S. grandis*.

### Trp metabolism response to heavy grazing

The most abundant and intensively studied endogenous auxin is IAA, which is essential for plant growth and development (Yue et al., 2014; Kasahara, 2016). In the Trp metabolism pathway, Trp is a precursor of auxin and various secondary metabolites (Maeda and Dudareva, 2012). The IAA is mainly synthesized by Trp-dependent and Trp-independent pathways (Mano and Nemoto, 2012). In the Trp-dependent pathway, IAA can be derived from the following pathways: Trp, IAM and IAA in the IAM pathway; Trp, indole-3-pyruvic acid (IPA), indole-3-acetaldehyde (IAAld) and IAA in the IPA pathway; Trp, indole-3-acetaldoxime (IAOx), IAN or IAAld, and IAA in the IAOx pathway; and Trp, TAM, *N*-hydroxytryptamine, IAOx, IAAld, and IAA in the TAM pathway (Woodward and Bartel, 2005; Mano and Nemoto, 2012). In the present study, TAM, *N*-hydroxytryptamine, and IAN showed elevated accumulation in heavy-grazing-induced dwarfed plants compared with NG plants, but the IAA content showed no obvious difference between the two groups. In addition, the expression level of AMI1, which converts IAM to IAA, was decreased in dwarfed plants. Fine-tuning of cellular auxin contents is important for plant survival (Casanova-Saez and Voss, 2019). We hypothesized that auxin contents are fine-tuned in heavily grazed plants and may play important roles in the regulation of plant development and responses to environmental stresses. In further studies, it will be important to explore the cross-talk of IAA with other phytohormones in the regulation of heavy grazing response.

## Data availability statement

The mass spectrometry proteomics data presented in the study are deposited in the ProteomeXchange Consortium (http://proteomecentral.proteomexchange.org) via the iProX partner repository, accession number PXD035914. The transcriptomic data presented in the study are deposited in the NCBI Sequence Read Archive (SRA) repository, accession number PRJNA867365.

## Author contributions

DW and YD conceived and designed the experiments. DW and YW performed the experiments. DW, YW, TZ, and RW analyzed the data. DW drafted the manuscript. All authors contributed to the article and approved the submitted version.

## Funding

This research was funded by the Natural Science Foundation of Inner Mongolia Autonomous Region (2020ZD06 and 2022LHMS03007), Key technology Project of Science and Technology Plan in Inner Mongolia Autonomous (2019GG009), Major special projects of science and technology in Inner Mongolia Autonomous Region (2019ZD008), and the Central Public-interest Scientific Institution Basal Research Fund (1610332020004).

## Conflict of interest

The authors declare that the research was conducted in the absence of any commercial or financial relationships that could be construed as a potential conflict of interest.

## Publisher’s note

All claims expressed in this article are solely those of the authors and do not necessarily represent those of their affiliated organizations, or those of the publisher, the editors and the reviewers. Any product that may be evaluated in this article, or claim that may be made by its manufacturer, is not guaranteed or endorsed by the publisher.

## Footnotes

^1^ http://www.genome.jp/kegg/

^2^ www.geneontology.org

^3^ http://www.ncbi.nlm.nih.gov

^4^ http://www.expasy.ch/sprot

^5^ https://www.uniprot.org/uniprotkb?facets=reviewed%3Afalse&query=%2A

^6^ https://www.ncbi.nlm.nih.gov/COG/

^7^ http://www.metwarebio.com/list/27.html

^8^ https://www.r-project.org/
