# Supplementary material for: Multi-omics analysis reveals the molecular changes accompanying heavy-grazing-induced dwarfing of Stipa grandis
Source: Front Plant Sci. 2022 Oct 4;13:995074. doi: 10.3389/fpls.2022.995074 (PMC9673880; doi:10.3389/fpls.2022.995074)
Supplement: Supplementary file 8 [file Data_Sheet_2.pdf]

Supplementary Figures

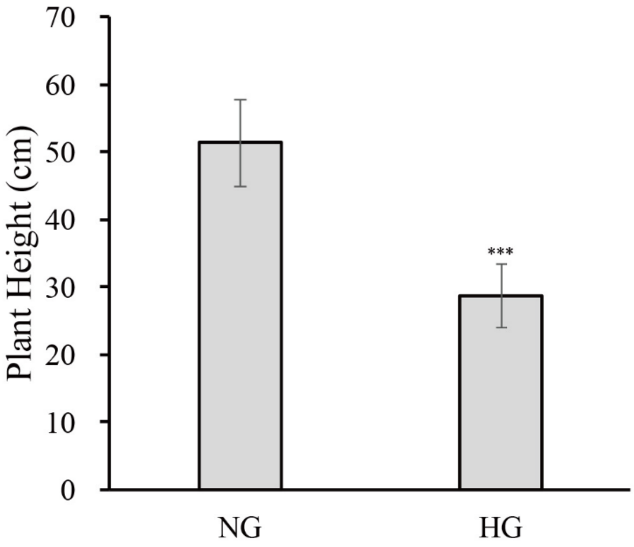

Supplementary Figure S1. Plant height of *Stipa grandis* under non-grazed (NG) and heavy-grazing (HG) treatments.

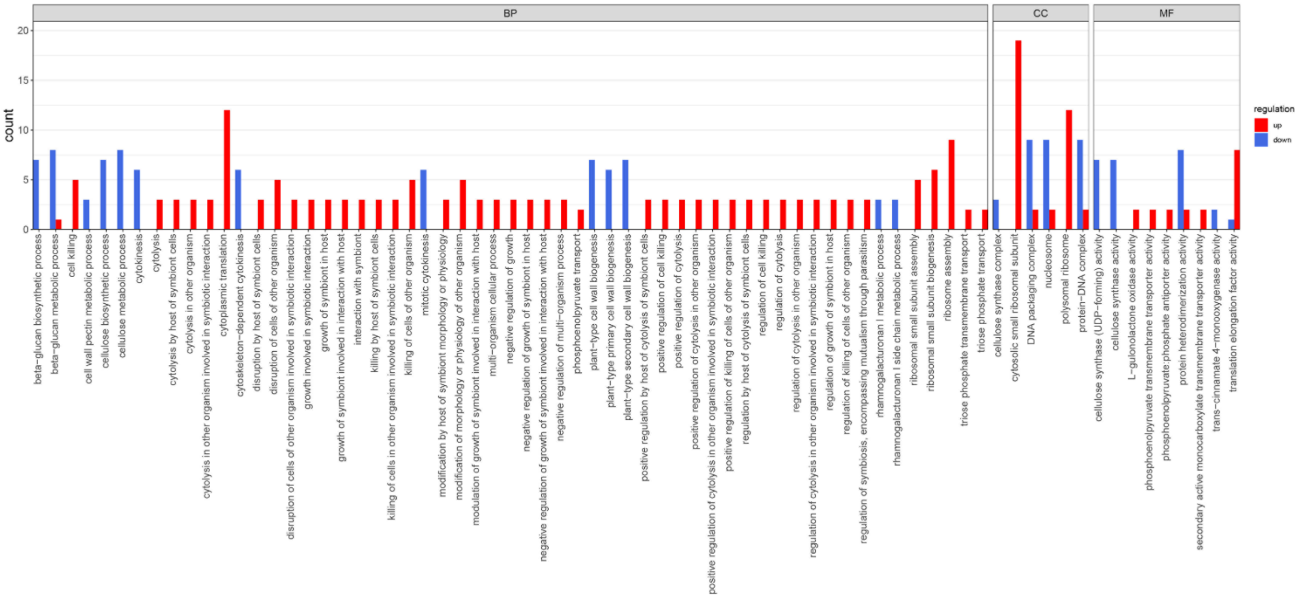

Supplementary Figure S2. Significantly enriched GO terms among differentially expressed proteins (DEPs).

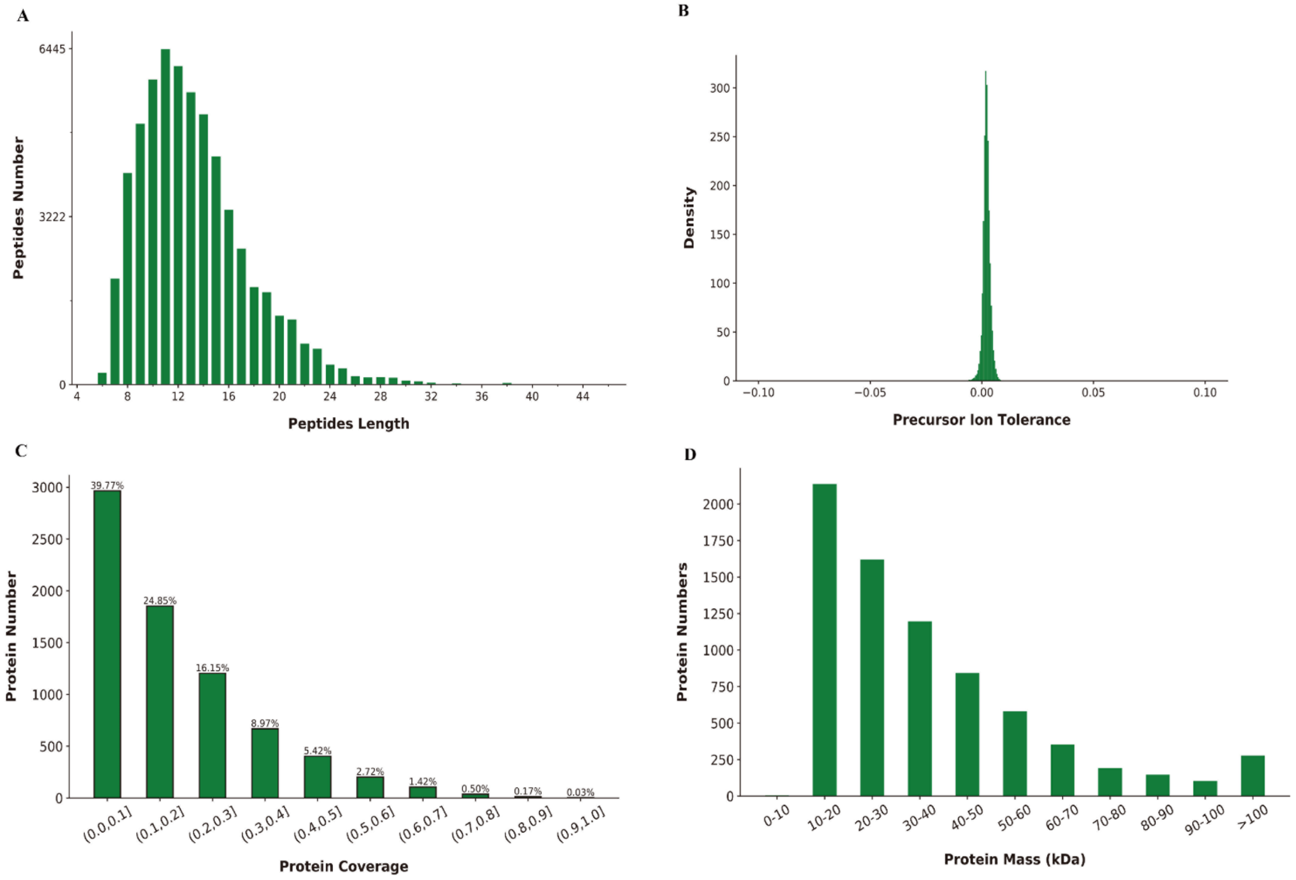

Supplementary Figure S3. Quality control of proteome profile. **(A)** Peptides length. **(B)** Precursor ion tolerance. **(C)** Protein coverage. **(D)** Protein mass.

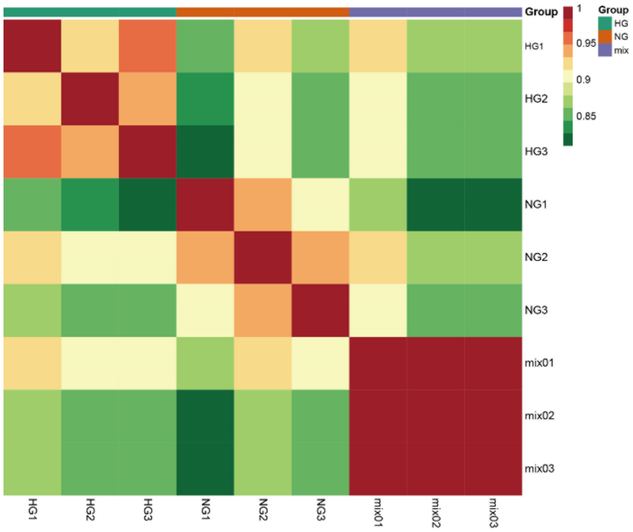

Supplementary Figure S4. Pearson's correlation analysis of intragroup correlation among metabolites in each NG and HG.

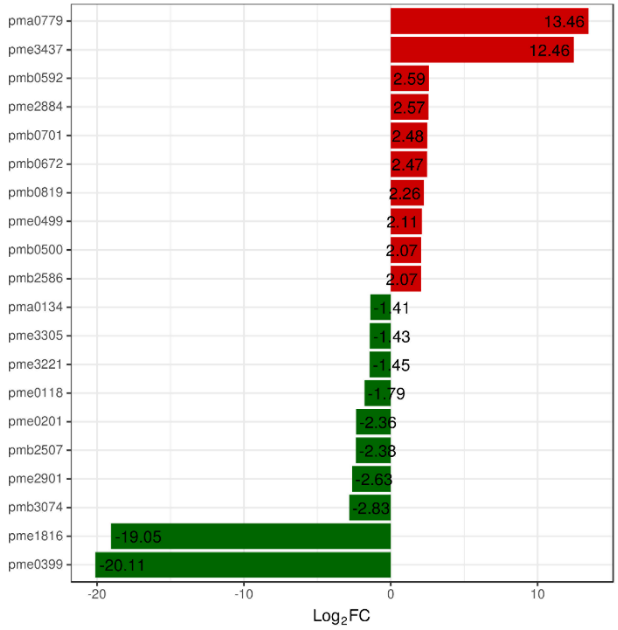

Supplementary Figure S5. The top 20 differentially accumulated metabolites (DAMs).

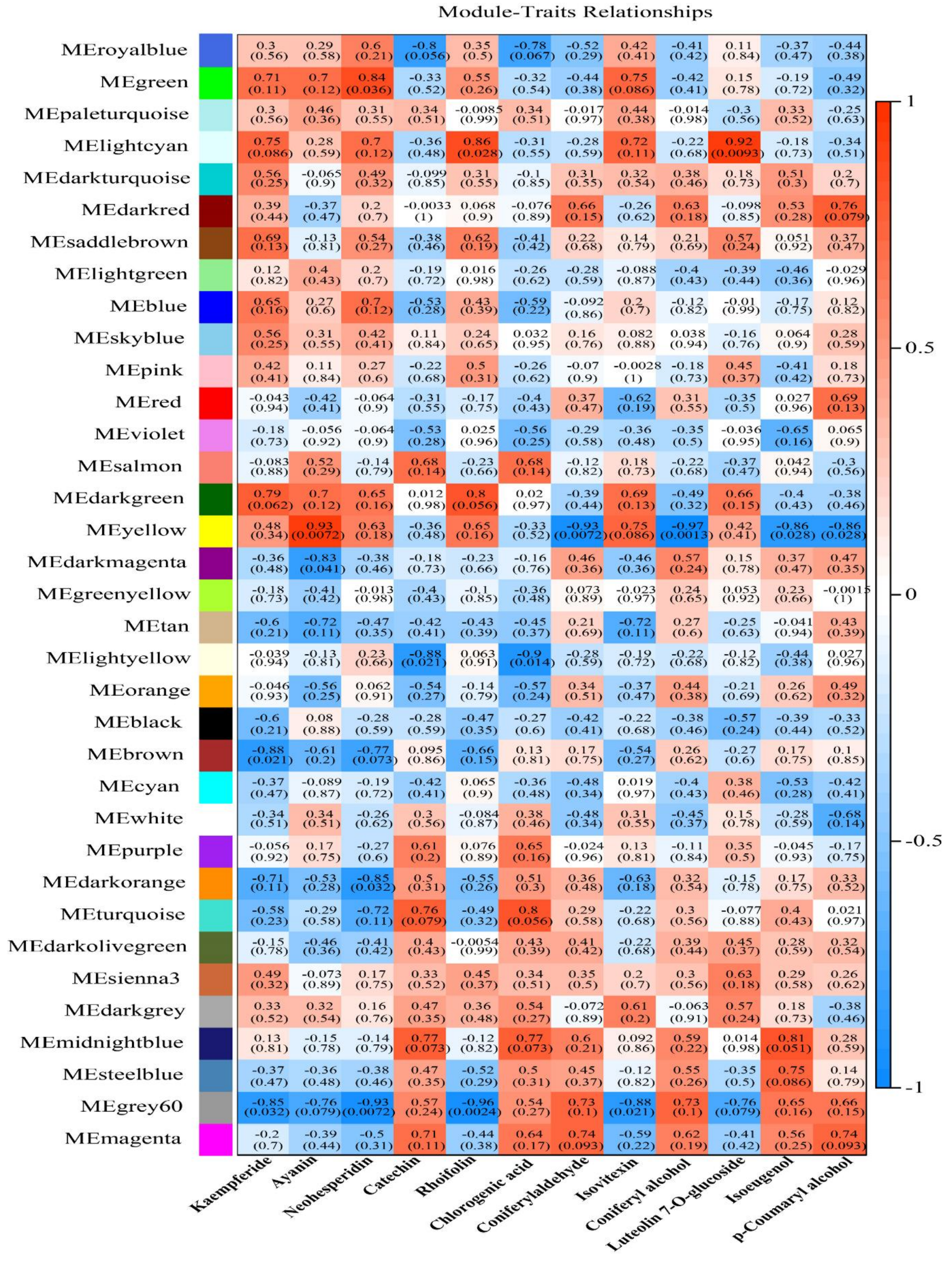

Supplementary Figure S6. Weighted gene co-expression network analysis (WGCNA) of co-expression genes with DAMs enriched in phenylpropanoid biosynthesis. Upper and lower values indicate the correlation coefficients and *p*-values, respectively.

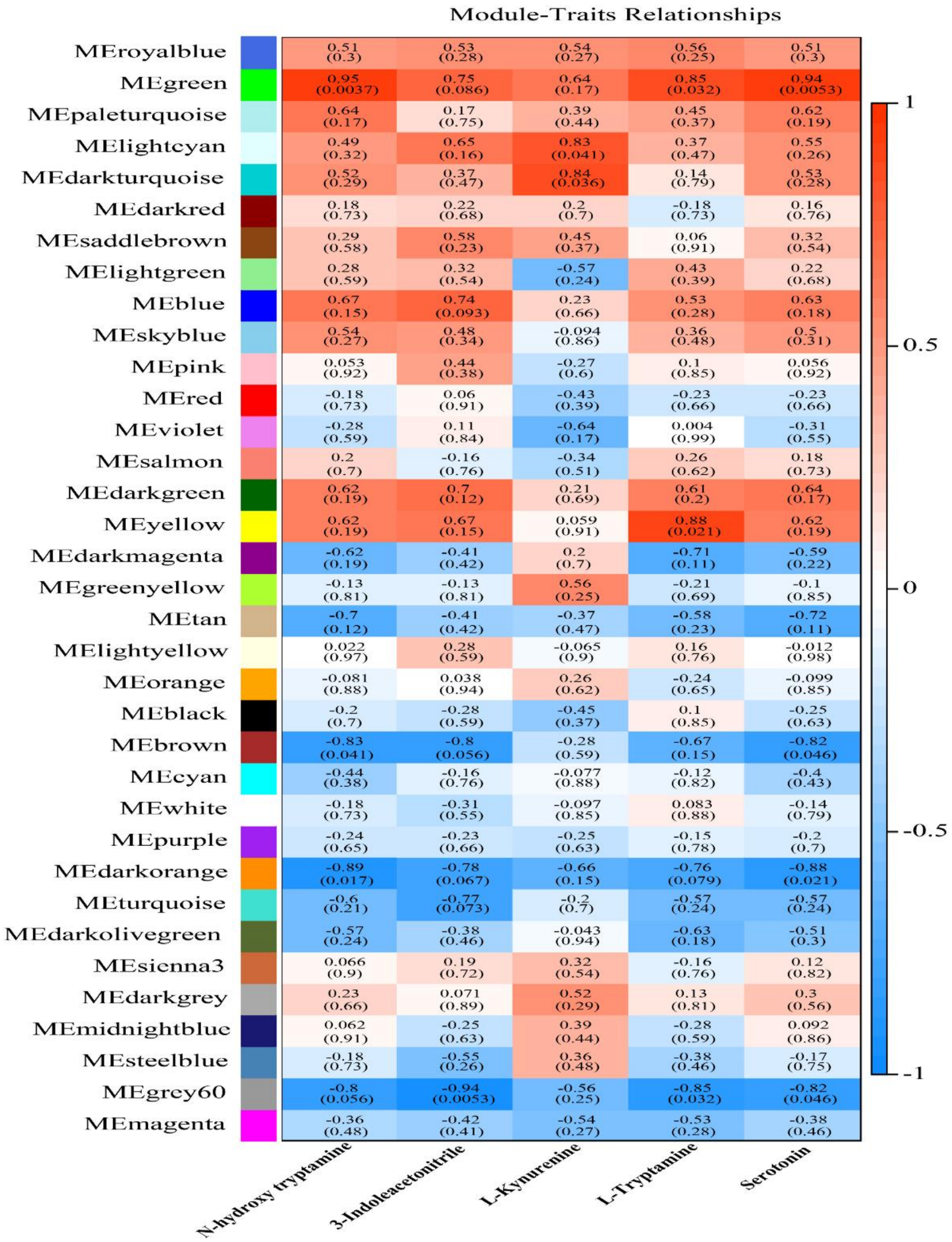

Supplementary Figure S7. WGCNA of co-expression genes with DAMs enriched in Trp metabolism. Upper and lower values indicate the correlation coefficients and *p*-values, respectively.
